# The mitogenome of *Pseudocrossidium replicatum*, a desiccation-tolerant moss

**DOI:** 10.1080/23802359.2020.1774436

**Published:** 2020-06-05

**Authors:** Miguel A. Cevallos, Gabriela Guerrero, Selma Ríos, Analilia Arroyo, Miguel Angel Villalobos, Helena Porta

**Affiliations:** a Centro de Ciencias Genómicas, Programa de Genómica Evolutiva, Universidad Nacional Autónoma de México, Cuernavaca, Morelos, C.P., México; b Centro de Investigación en Biotecnología Aplicada, Instituto Politécnico Nacional. Tepetitla de Lardizábal, Tlaxcala, C.P., México; c Instituto de Biotecnología, Departamento de Biología Molecular de Plantas, Universidad Nacional Autónoma de México, Morelos, C.P., México

**Keywords:** Bryophytes, Pottiaceae, mitochondria, chondriome, next-generation sequencing

## Abstract

Bryophytes are the earliest plant group on Earth. They are a fundamental component of many ecosystems around the World. Some of their main roles are related to soil development, water retention, and biogeochemical cycling. Bryophytes include liverworts, hornworts, and mosses. The sequencing of chloroplast and mitochondria genomes has been useful to elucidate the taxonomy of this heterogeneous plant group. To date, despite their ecological importance only 41 mosses mitogenomes have been deposited in the GenBank. Here, the complete mitochondria genome sequence of *Pseudocrossidium replicatum*, a moss of the Pottiaceae family isolated in Tlaxcala, Mexico, is reported. The mitochondrial genome size of *P. replicatum* comprises 105,495 bp and contains the groups of genes described for other bryophytes mitogenomes. Our phylogenetic analysis shows that during the evolution of the mosses’ mitogenome, *nad7*, *rps4*, *rpl16*, and *rpl10* genes were lost independently in several lineages. The complete mitogenome sequence reported here would be a useful tool for our comprehension of the evolutionary and population genetics of this group of plants.

A crucial step in the evolution of the plant kingdom was the transition from aquatic to terrestrial environments. The bryophytes is one of the groups of land plants that emerged from this transition. Fossil evidence and molecular dating indicate that bryophytes appeared during the Ordovician period (Wellman et al. 2003; Clarke et al. 2011; Magallon et al. 2013). The bryophytes, composed of mosses, liverworts, and hornworts, are the earliest terrestrial plants that exist on Earth. Their success relies on adaptations to survive in the new habitat. In general, the structure of bryophytes consists of a haploid and photosynthetic gametophyte protected with a waxy coating on their shoots, multicellular structures (antheridia and archegonia) that protect gametes from desiccation; and, a diploid unbranched sporophyte containing a single sporangium attached to the gametophyte.

Bryophytes are a fundamental component of many ecosystems around the World. They play a crucial role in soil development, water retention and biogeochemical cycling, among many others (Sun et al. 2013). Bryophytes embrace about 21,000 species; within them, mosses comprise between 7000–13,000, liverworts 5000–7500, and hornworts about 215 (Budke et al. 2018). The taxonomy of this heterogeneous group is complex and, for this reason, molecular tools and a wider knowledge of their phylogenetic relationships are always welcomed.

Chloroplast and mitochondria genomes have been used for these purposes because these organelles have low-frequency genetic recombination and a uniparental mode of inheritance. Despite the importance of bryophytes in general and mosses in particular, only 41 mitogenomes have been deposited in the GenBank.

To expand our understanding of these primitive land plants in this work, we report the complete mitogenome sequence of the moss *Pseudocrossidium replicatum* that belongs to the moss family Pottiaceae and can be found in the central highlands of México, South America, and the southern USA (Zander 1993).

In January 2008, we collected a specimen of *P. replicatum* in Ixtacuixtla, Tlaxcala, México (19-20-03.3 N, 98-21-59.9 W, 2159 μ.a.s.l), and implemented an *in vitro* culture of this exemplar starting from a single spore. This culture has been maintained since then, through weekly subcultures in PpNH_4_ culture medium, in a growth chamber at 23 °C and 30% of relative humidity, with a photoperiod of 16/8 h light/dark with a light intensity of 55 μmol photons m^−2^s^−1^ (Cevallos et al. 2019). Genomic DNA was isolated from 7 days-old protonemata cultivated *in vitro* using The Manual ZR Plant-Seed DNA Microprep kit (ZymoResearch). A DNA pair-end library (PE 2 × 75) from Illumina NextSeq500 platform was prepared and sequenced at Unidad Universitaria de Secuenciación Masiva de DNA de la Universidad Nacional Autónoma de México. Reads were assembled with SPAdes-3.9.0. From the assembly, we extracted a single contig with partially overlapping ends, embracing the complete mitochondrial genome of *P. replicatum*.

The mitochondrial genome size of *P. replicatum* comprises 105,495 bp and falls within a range size from 100,342 bp from *Mielichhoferia elongata* to 115,146 bp from *Atrichum angustatum*.

The *P. replicatum* mitogenome contains the groups of genes described for other bryophytes mitogenomes: 3 rRNAs genes, 24 tRNAs genes and 41 open-reading frames (ORFs) for functional proteins previously described that include 10 genes for the small subunit ribosomal proteins, 5 genes for proteins of the large ribosomal subunit, 9, 2, one, 3, and 5 genes for the mitochondrial respiratory chain complexes I, II, III, IV, and V, respectively, 4 genes involved in cytochrome c biogenesis, one gene for the *sec-independent protein*, and one *apocytochrome b* gene. A group II intron-associated open-reading frame is present in *cox1* intron number 2 codifying for a hypothetical reverse transcriptase-maturase. All the moss sequences analyzed in this work contain this putative protein although in several of them it was not annotated.

To determine the relationships between the analyzed mosses, a concatenated alignment was done with Clustal Omega and used to construct a maximum likelihood phylogenetic tree (bootstrap of 1000) with MEGAX (Sievers and Higgins 2014; Kumar et al. 2016). The tree include 16 proteins (*atp1*, *atp4*, *atp6*, *atp8*, *atp9*, *cox1*, *cox2*, *cox3*, *nad1*, *nad2*, *nad3*, *nad5*, *nad9*, *rps12*, *rps13*, and, *rps19*) from 26 different genera of mosses considering only a member of each genera: *Anomodon attenuates* (NC_021931.1), *Atrichum angustatum* (NC_024520.1), *Bartramia pomiformis* (NC_024519.1), *Brachythecium rivulare* (NC_031212.1), *Bucklandiella orthotrichacea* (NC_026974.1), *Buxbaumia aphylla* (NC_024518.1), *Chionoloma tenuirostre* (NC_028040.1), *Climacium americanum* (NC_024515.1), *Codriophorus aciculare* (NC_026784.1), *Funaria hygrometrica* (NC_024523.1), *Hypnum imponens* (NC_024516.1), *Mielichhoferia elongate* (NC_036945.1), *Nyholmiella gymnostoma* (NC_031391.1), *Orthotrichum bicolor* (NC_031389.1), *Physcomitrella patens* (NC_007945.1), *Polytrichum commune* (NC_039775.1), *Pseudocrossidium replicatum* (MT310681), *Ptychomnion cygnisetum* (NC_024514.1), *Racomitrium emersum* (NC_026975.1), *Sanionia uncinata* (NC_027974.1), *Sphagnum palustre* (NC_024521.1), *Stoneobryum bunyaense* (NC_031392.1), *Syntrichia filaris* (NC_027515.1), *Tetraphis pellucida* (NC_024290.1), *Tetraplodon fuegianus* (NC_028191.1), *Ulota crispa* (NC_031393.1). The fern *Ophioglossum californicum* (NC_030900) was used as an out-group. Figure 1 shows that the most basal mitochondrial moss is *S. palustre* and the closest relative to *P. replicatum* in *S. filaris*. Our analysis indicates that genes *nad7*, *rps4*, *rpl16*, and *rpl10* have been lost independently in several moss lineages. The mitogenome of *P. replicatum w*ill be useful to enrich the future analysis of the evolution of bryophytes.

**Figure 1.**
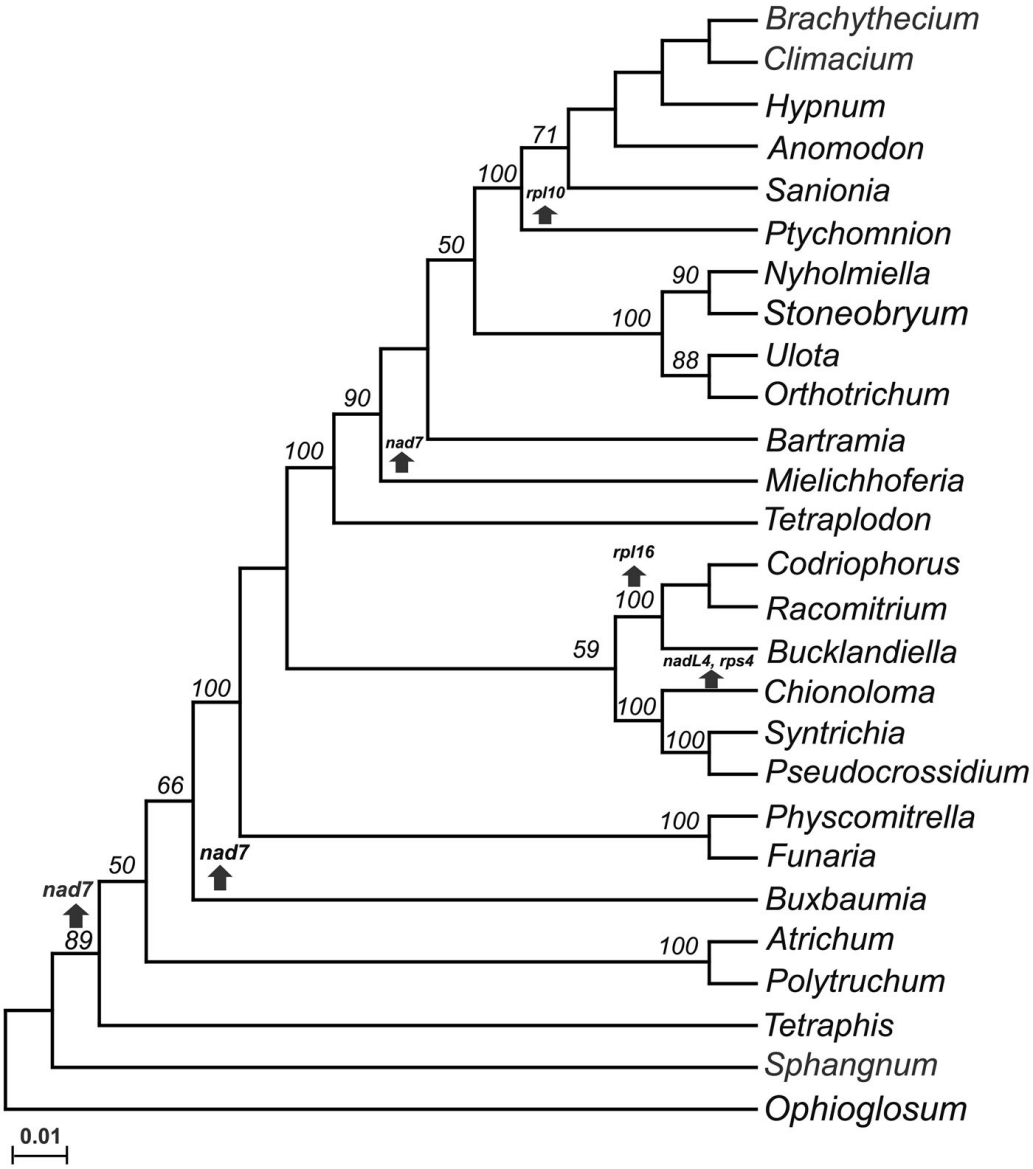
Mosses mitochondrial genomes cladogram. A maximum likehood tree was constructed using a concatenated alignment of proteins (*atp1*, *atp4*, *atp6*, *atp8*, *atp9*, *cox1*, *cox2*, *cox3*, *nad1*, *nad2*, *nad3*, *nad5*, *nad9*, *rps12*, *rps13*, and, *rps19*) from 26 mosses: *Anomodon attenuates* (NC_021931.1), *Atrichum angustatum* (NC_024520.1), *Bartramia pomiformis* (NC_024519.1), *Brachythecium rivulare* (NC_031212.1), *Bucklandiella orthotrichacea* (NC_026974.1), *Buxbaumia aphylla,* (NC_024518.1), *Chionoloma tenuirostre* (NC_028040.1), *Climacium americanum* (NC_024515.1), *Codriophorus aciculare* (NC_026784.1), *Funaria hygrometrica* (NC_024523.1), *Hypnum imponens*, (NC_024516.1), *Mielichhoferia elongate* (NC_036945.1), *Nyholmiella gymnostoma* (NC_031391.1), *Orthotrichum bicolor* (NC_031389.1), *Physcomitrella patens* (NC_007945.1), *Polytrichum commune* (NC_039775.1), *Pseudocrossidium replicatum* (MT310681), *Ptychomnion cygnisetum* (NC_024514.1), *Racomitrium emersum* (NC_026975.1), *Sanionia uncinata* (NC_027974.1), *Sphagnum palustre* (NC_024521.1), *Stoneobryum bunyaense* (NC_031392.1), *Syntrichia filaris* (NC_027515.1), *Tetraphis pellucida* (NC_024290.1), *Tetraplodon fuegianus* (NC_028191.1), *Ulota crispa* (NC_031393.1). The mitochondrial genome sequence from the fern *Ophioglossum californicum* was used as out-group. Numbers indicate bootstrap values. Arrows pointing up indicate the loss of genes.

## Data Availability

The data supporting the findings of this study are available in GenBank of NCBI at https://www.ncbi.nlm.nih.gov, reference number MT310681.
